# Adherence and contamination in a 1‐year physical activity program in childhood cancer survivors: A report from the SURfit study

**DOI:** 10.1002/cam4.6096

**Published:** 2023-05-18

**Authors:** Ruedi Jung, Simeon J. Zürcher, Christina Schindera, Julia Braun, Wei Hai Deng, Nicolas X. von der Weid, Corina S. Rueegg, Susi Kriemler

**Affiliations:** ^1^ Epidemiology, Biostatistics and Prevention Institute University of Zurich Zurich Switzerland; ^2^ Center for Psychiatric Rehabilitation University Hospital for Mental Health, University of Bern Bern Switzerland; ^3^ Pediatric Oncology/Hematology University Children's Hospital Basel Basel Switzerland; ^4^ Institute of Social and Preventive Medicine University of Bern Bern Switzerland; ^5^ Oslo Centre for Biostatistics and Epidemiology Oslo University Hospital Oslo Norway

**Keywords:** adherence, childhood cancer survivors, contamination, exercise, physical activity

## Abstract

**Purpose:**

Meeting intervention requirements is crucial in behavioral trials. We examined patterns and predictors of physical activity (PA) adherence and contamination in a 1‐year individualized randomized controlled PA behavioral intervention in childhood cancer survivors (CCS).

**Methods:**

CCS aged ≥16 at enrolment, <16 at diagnosis, and ≥5 years in remission were identified from the Swiss Childhood Cancer Registry. We asked participants randomized to the intervention group to perform an additional ≥2.5 h of intense PA/week and controls to continue as usual. Adherence to the intervention was assessed by online diary (adherent if ≥2/3 of individual PA goal reached) and contamination for the control group by pre‐ and post‐questionnaire including PA levels (contaminated if >60 min increase/week in PA). Predictors of adherence/contamination including quality of life (36‐Item Short Form Survey) were assessed by questionnaire. We used logistic (control group) and mixed logistic regression models (exercise group) to estimate predictors of study adherence and contamination.

**Results:**

One hundred and forty‐four survivors (30.4 ± 8.7 years old, 43% females) were included. Adherence was 48% (35/73) in the intervention group, while 17% (12/71) of controls contaminated group allocation. Predictors for PA adherence were female sex (OR 2.35, *p* = 0.03), higher physical (OR 1.34, *p* = 0.01) and mental quality of life (OR 1.37, *p* = 0.001), and week into the intervention (OR 0.98, *p* < 0.001). Clear differences in PA behavior of adherent and non‐adherent participants were seen from week four. No significant predictors for contamination were found for controls.

**Conclusion:**

Adherence to PA behavior interventions remain challenging in both groups. Further long‐term trials should consider intense motivational support within the first month, more detailed data collection for the control group, adjustments to power calculations and other study designs to minimize non‐adherence and contamination.

## Graphical abstract

We examined patterns and predictors of physical activity (PA) adherence and contamination in a one‐year individualized randomized controlled PA behavioral intervention in childhood cancer survivors (CCS). Adherence was 48% in the intervention group, while 17% of controls contaminated group allocation. Clear differences in PA behavior of adherent and non‐adherent participants were seen from week 4. Further long‐term trials should consider intense motivational support within the first month, adjustments to power calculations and other study designs to minimize non‐adherence and contamination.

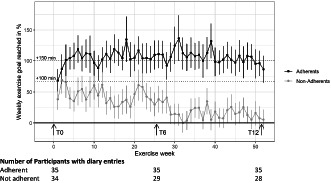

## 1 INTRODUCTION

Physical activity (PA) is generally known to be associated with improved overall health including reduced all‐cause mortality, improved cardiovascular health, and lower risk of developing cancer.^1^ Childhood cancer survivors (CCS) who underwent intensive cancer treatment with chemotherapy, radiotherapy, hematopoietic stem cell transplantation, or surgery frequently experience delayed effects such as fatigue, pain, overweight, and depression, which negatively influence their fitness. As such, reduced PA levels often persist long after cancer therapy, potentially aggravating late effects of cancer treatment such as cardiovascular diseases, metabolic syndrome, osteoporosis, fatigue, or cognitive decline.^2^ These adverse effects could be prevented, or at least reduced, if cancer survivors stayed as active as possible, as recommended by international PA guidelines.^1^, ^3^

Many trials to increase PA are ineffective and show null results.^4^, ^5^ Obvious reasons may be that those in the intervention group do not fully implement the agreed program and/or the control group does increase their PA although they should not.^6^ The extent to which a person's behavior corresponds with agreed recommendations from a health care provider is defined as adherence and is reported as challenging both during and after cancer treatment.^1^, ^7^, ^8^ It is crucial to interpret efficacy and clinical relevance of randomized controlled trials (RCTs) with adherence issues in hand.^1^, ^7^ However, adherence to the prescribed PA program is often either poorly reported or not reported at all in RCTs, and most research focuses on the intervention (e.g., increase PA) rather than the control group (e.g., no increase in PA).^7^, ^8^, ^9^, ^10^ Only a few studies have focused on identifying predictors to understand adherence and contamination in behavioral trials of cancer survivors, and have revealed conflicting results for sociodemographic, clinical, psychosocial, and environmental predictors of exercise adherence.^1^

The aim of this study was to describe and identify predictors of exercise adherence and contamination of the intervention and control group within an RCT aiming to increase PA over 1 year in adult CCS participating in the SURfit study.^11^

## 2 METHODS

### 2.1 Trial design and participants

We used data from the SURfit study (ClinicalTrials.gov identifier: NCT02730767),^11^ a two‐armed, parallel‐group, open‐label RCT comprising a 1‐year PA intervention. Eligible CCS were aged ≥16 years at enrolment, <16 at diagnosis, and ≥5 in remission, identified through the Swiss Childhood Cancer Registry. All included CCS were treated at a Swiss Paediatric Oncology clinic and suffered from a cancer classifiable according to the International Classification of Childhood Cancer^12^ or Langerhans Cell Histiocytosis. Detailed inclusion/exclusion criteria are described by Rueegg et al. (2017).^11^ Briefly, all CCS could participate if willing to change PA, except those that already performed intense PA beyond 4 h weekly. Eligible CCS were contacted by letter between June 2015 and February 2018. Interested CCS were 1:1 randomized into the intervention and control arms using web‐based minimization randomization by a person independent from recruitment. Stratification factors were sex and the four cancer categories (leukemia/lymphoma, central nervous system tumors, bone tumors/soft tissue sarcomas, and other tumor diagnoses). This single‐center trial was conducted at the University Children's Hospital Basel in Switzerland between September 2015 and February 2019. The primary aim of SURfit was to assess the effect of a PA intervention on cardiovascular health in CCS. Study appointments took place at baseline (T0), after 3 (T3), 6 (T6), and 12 months (T12). Participants who dropped out due to a reason not related to the study (e.g., moving abroad, pregnancy) were excluded from the current analyses. The study was authorized by the Swiss Ethics Committee of Northwest and Central Switzerland and all participants gave written informed consent.

### 2.2 Intervention and control conditions

Participants of the intervention group were asked to add ≥2.5 h of intense PA/week to their baseline activity level. Intense PA was reached when participants had a fast breathing/heartbeat for at least 20 min. Together with a physiotherapist, individualized PA programs were developed and self‐implemented into each survivor's daily living, aiming to reach a plus of 2 h aerobic and 0.5 h strength building PA per week. The PA program was not supervised except for follow‐up contacts with the physiotherapist described below. The intervention participants increased their target PA from week one. We used the following motivational tools to achieve optimal adherence: Regular contact with the physiotherapist (face‐to‐face at 0, 3, 6, and 12 months, and phone calls after 1, 2, 4, 5, 8, and 10 months), pedometers (daily reporting of steps), and a self‐administered web‐based daily activity diary with immediate graphical feedback. Diary entries were monitored, and participants reminded to complete missing entries each week. Participants of the control group were asked to keep their PA levels constant.

### 2.3 Outcome: Adherence and contamination

PA levels of the intervention group were assessed by a web‐based diary filled out on daily basis. Automated reminders were sent out by email, and if participants were not responsive within 3 days, phone calls were initiated. In the diary, intervention participants registered each sport session performed, the duration and type of PA performed. The types of activities were manually grouped into jogging/bicycling/swimming, fitness/gym, team sports, hiking/winter sports, and other activities. PA of the control group was assessed by a 7‐day recall questionnaire filled out by the participant at baseline and at the 12‐month assessment. Answers were verified and discussed by an exercise specialist during an interview. It was specifically emphasized to have a typical week reported within the last 3 months. As the controls were allowed to switch to the exercise program after the trial, the 12‐month interview was taken as baseline assessment of their PA behavior to receive the same personalized PA counseling with motivational tools as the intervention group, but without personal follow‐up coaching.

We defined participants of the intervention group to be adherent if they reached ≥2/3 of their individual intense PA goal (100 min of the agreed 150 min additional PA/week) based on the web‐based diary and referred to them as “exercise adherent.” For this, all cumulative PA hours over the 1 year were considered and a binary variable was built to determine whether a participant was adherent or not for each week. For the regression model, we used the percentage of weeks where the PA goal was reached as outcome. Days with no PA entries were set to 0 min PA suggesting that no sport was performed. Control group participants were defined as “control contaminated” if they reported >60 min increase in intense PA/week based on the questionnaire and confirmed by an interview at T0 and T12 done by the coach.^11^ Dropouts were classified as non‐adherents for the yearly goal reached, but were included in the prediction model until withdrawal for the intervention group.

### 2.4 Predictors

Predictors of adherence were not an a priori research question and were thus selected from our available data and a literature review, and on their ease to be assessed in clinical practice through simple questions at the beginning of an intended increase in PA.^7^, ^10^, ^13^ Sex and body mass index (BMI) were recorded at baseline. BMI was dichotomized into being overweight (yes, no) with a cutoff of 25 kg/m^2^. Having a partner, quality of life (Short Form 36, SF‐36^14^), fatigue (Checklist Individual Strength, CIS^15^), intense PA hours at baseline, and having a physically demanding job (e.g., a profession with physical work of at least moderate intensity over several hours a day^16^) were assessed through self‐reported questionnaires at each time point. The SF‐36 is a validated quality of life questionnaire consisting of 36 questions that are aggregated in subscales and a Physical (PCS) and a Mental Component Summary (MCS) based on z‐scores using age‐ and sex‐stratified data from a Swiss norm population (*N* = 1209).^14^ The CIS is a validated 20‐item questionnaire that is designed to measure four aspects of fatigue that may have been experienced during the previous 2 weeks, that is severity of fatigue, concentration, motivation, and physical activity. *Z*‐scores for CIS were calculated based on a Dutch norm population.^15^

### 2.5 Statistical analyses

We calculated the average PA h/week and the percentage of individual goal reached for the intervention group, overall and stratified for adherent/non‐adherent participants over the year, and for the first and second half of the 1‐year intervention period. We investigated the average number/duration of each PA session, the type of sport performed; and the predictors mentioned above. We used two separate models to look at associations (predictors) for contamination in controls and adherence in intervention participants, respectively. Predictors of contamination within the control group were investigated using a multivariable logistic regression model (increase T0/T12). Predictors of adherence within the intervention group were investigated with a mixed effects logistic regression (with repeated measures of weekly adherence as yes/no outcome) with random intercept and updated time‐varying covariates where available (T3: BMI, CIS; T6: BMI, PCS, MCS, CIS). For dropouts, observations until withdrawal were included in the analysis. R (v4.0.2, R Foundation) software was used with the packages lme4, ggplot2, and dplyr.

## 3 RESULTS

From a total of 1450 eligible CCS, 842 got invited for study participation whereof 151 (18%) were randomly assigned to one of the two treatment arms with 76 intervention and 75 control group participants. From those, seven participants were not included in the current analysis due to dropout reasons unrelated to the study. From 144 included CCS, 132 (92%) completed the study; 10 intervention and 2 control group participants dropped out (see Table 1; Figure S1). Baseline characteristics of these 17‐ to 49‐year‐old CCS (mean 30 ± 9 years) were well matched between groups.

**TABLE 1:** Baseline characteristics of the study population (*n* = 144).

	Intervention	Control
	(*n* = 73)	(*n* = 71)
Basic characteristics		
Sex (female)	31 (42%)	31 (44%)
Age at study (year)	31.6 (8.5)	29.3 (8.8)
Height (cm)	170.0 (8.8)	171.4 (9.7)
Weight (kg)	71.2 (15.4)	69.2 (13.8)
BMI (kg/m^2^)	24.5 (4.4)	23.4 (3.6)
BMI ≥25 kg/m^2^	31 (42%)	18 (25%)
Cancer related information		
Age at diagnosis (year)	7.5 (5.1)	7.5 (4.7)
Time since diagnosis (year)	24.1 (8.7)	21.9 (9.2)
ICCC‐3 cancer diagnoses		
I Leukemia	23 (32%)	29 (41%)
II Lymphoma	17 (23%)	14 (20%)
III Central nervous system tumor	11 (15%)	5 (7%)
IV–XIII other tumors	22 (30%)	23 (32%)
Socioeconomic conditions		
Married/partnership (yes)	39 (53%)	37 (52%)
with children (yes)	13 (18%)	11 (15%)
Education		
Compulsory school	2 (3%)	8 (11%)
Apprenticeship	38 (52%)	39 (56%)
Higher education	33 (45%)	23 (33%)
Time at school, education, work >30 h	58 (81%)	58 (82%)
Physical job (yes)	19 (26%)	20 (28%)
Quality of life and fatigue		
Physical component summary (z‐score)	−0.1 (0.9)	0.1 (0.9)
Mental component summary (z‐score)	0.1 (1.0)	−0.2 (1.5)
Fatigue, checklist individual strength (z‐score)	0.3 (1.0)	0.2 (1.0)
Health related behaviors		
Intense physical activity at baseline (h/week)	1.0 (1.2)	1.6 (1.4)
Unhealthy nutrition (>2 days/week)^a^	35 (48%)	30 (44%)
Alcohol intake (≥3 times/week)	9 (12%)	7 (10%)
Smoker (yes)	19 (26%)	15 (21%)

*Note*: Data are presented as *n* (%)/mean (SD). Number of participants included (Intervention/Control): Education *n* = 73/70; Time at school, education, work *n* = 72/71; PCS & MCS *n* = 71/70; CIS *n* = 71/68.

Abbreviations: BMI, body mass index; ICCC‐3, international classification for childhood cancer third edition.

^a^ Less than 3 healthy portions per day (vegetables or fruits).

Overall, 17 (23%) intervention group participants reached 100% of their personalized goal (+2.5 h/week), while 35 (48%) adhered to the study program (≥2/3 of individual goal reached). Eight intervention participants dropped out in the first and two in the second half of the study. In the control group, 12 (17%) participants increased their weekly PA more than allowed (>60 min/week). Of those, 2 dropped out and 10 contaminated group allocation.

### 3.1 Details on adherence to the physical activity intervention

**FIGURE 1:**
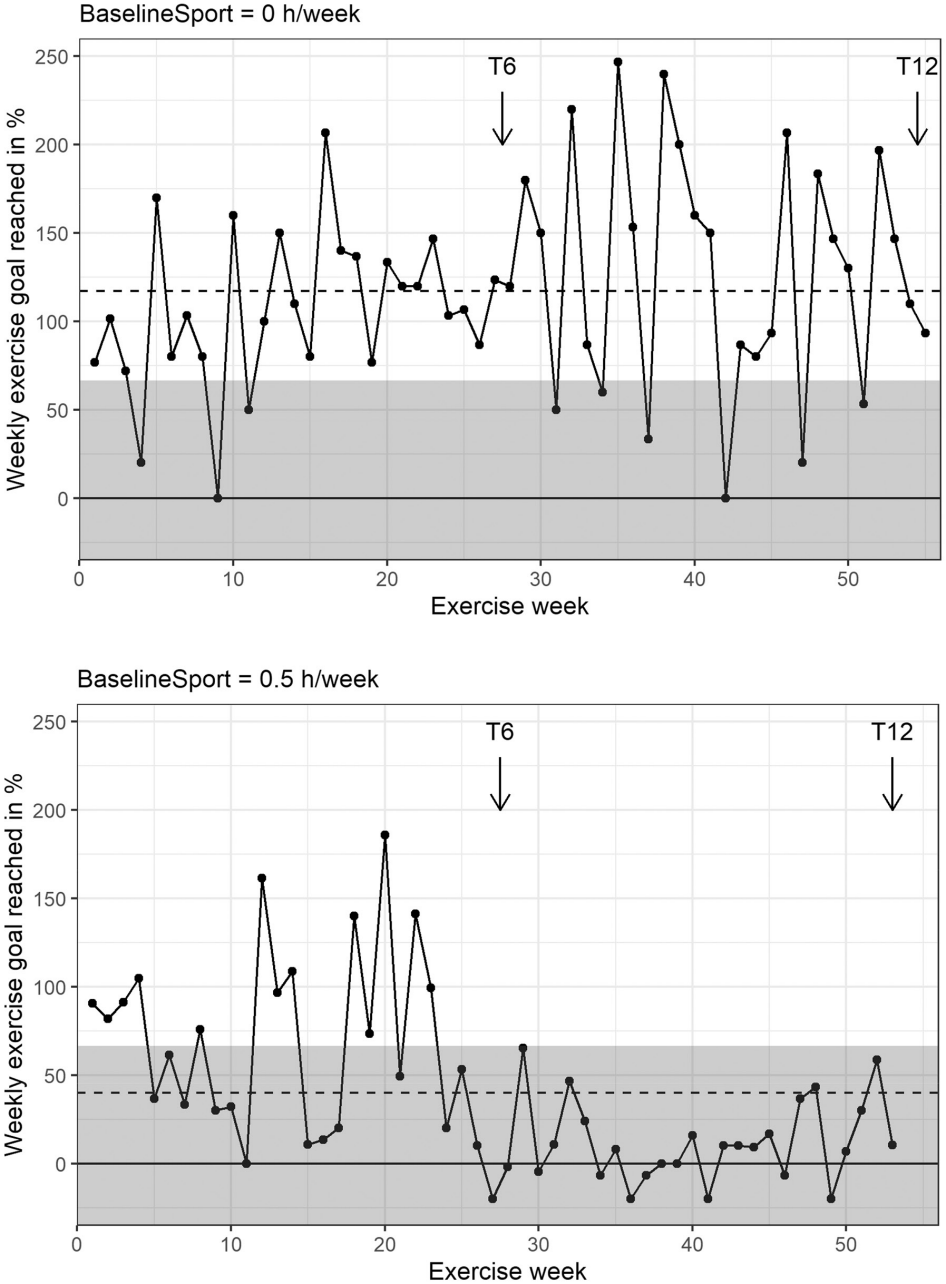
Percent of individual weekly physical activity goal reached based on self‐reported online diary entries. Accordingly, an example of an adherent (top) and non‐adherent (bottom) participant of the intervention group are displayed. Assessment periods at mid‐term (T6) and study end (T12) are highlighted and participants annual weekly mean of physical activity shown as dashed line. Gray areas highlight when the weekly goal was not reached (<2/3).

**FIGURE 2:**
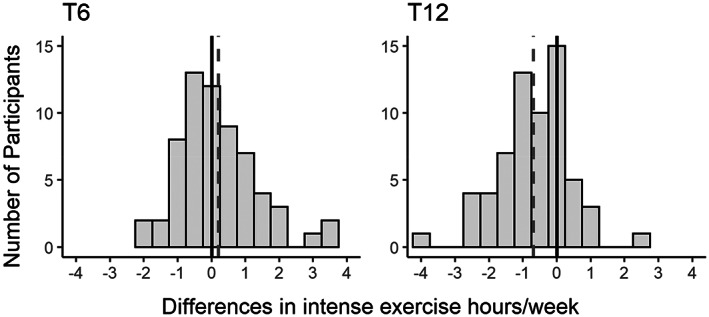
Distribution and mean (dashed line) of difference in individual weekly intense physical activity (PA) hours between the two assessments at mid‐term (T6) and study end (T12) and their annual PA average (0‐line). Positive values denote participants that were more active than their mean of PA.

Figure 1 shows the weekly proportion of personal PA goal reached of a random intervention adherent and a non‐adherent participant. Intervention group participants reached on average 72% of their individual PA goal. The number of PA adherent participants decreased from the first (53%, *n* = 39) to the second half (45%, *n* = 33) of the study. Figure 2 shows that participants were on average 12 min/week above their annual average at mid‐term (T6) and 42 min/week below at study end (T12).

**FIGURE 3:**
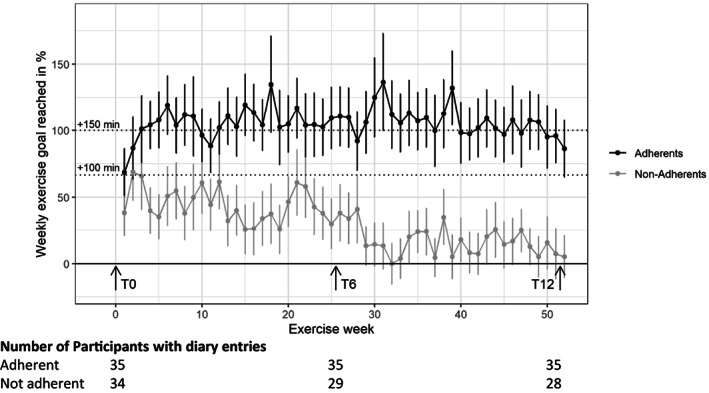
Tracking of adherence to physical activity (PA) during the study duration according to adherent/non‐adherent participants by diary entries. Values denote overall means and 95%‐CI. A clear difference among the PA behavior can be detected (95%‐CI did not overlap) from week 4 on. Participant's PA goal (+150 min) and threshold to be defined as adherent (+100 min) are highlighted. T0, baseline visit; T6, 6‐month follow‐up; T12, 12‐month follow‐up.

Intervention group participants reporting their PA behavior (*n* = 69, missing information *n* = 4) were engaged in an average of 2.5 h of weekly PA during the study. While PA adherents spent around 1 h above, non‐adherents spent 1 h below this weekly PA average. The favorite sports of intervention participants were jogging, bicycling, and swimming (48% of total 9177 PA hours), followed by fitness and gym (26%). The distribution of sport types was comparable in PA adherents/non‐adherents (Table 2). Tracking of PA behavior over time showed a striking picture (Figure 3): Non‐adherents reached only about 50% of their personal goal already at the very beginning from week 4 on, and further decreased their levels over time. PA adherents reached on average about 100% of their individual goal during the whole intervention period.

**TABLE 2:** Types of sport and physical activity behavior of participants of the intervention group according to adherence status.

Type of PA behaviour	Adherents	Non‐Adherents
	(*n* = 35)	(*n* = 34)
Type of sport		
Jogging, bicycling, swimming	47% (3055 h)	49% (1304 h)
Fitness, gym	26% (1713 h)	17% (465 h)
Team sports	11% (734 h)	17% (442 h)
Hiking, winter sports	14% (921 h)	12% (315 h)
Others	1% (84 h)	5% (144 h)
PA behavior		
Intense PA (h/week)	3.4 (2.9–3.9)	1.6 (1.2–2.0)
Number of PA sessions (*n*/year)	185 (164–205)	100 (76–124)
Time of each session (min/session)	62 (56–69)	58 (49–66)

*Note*: Data based on self‐reported data from the online diary. Data are presented as % (cumulative hours over the intervention period)/mean (95%‐CI).

### 3.2 Predictors of study adherence and contamination

Predictors of study adherence can be found in Table 3. For the intervention group, study adherence decreased over the year with a drop of roughly 2% per week. The odds of study adherence were 2.4 times higher in females than males. Higher physical and mental quality of life were associated with higher study adherence, leading to an 34% and 37% higher adherence for each standard deviation (corresponding to 1 z‐score unit), respectively. There was no evidence of an effect of being obese, having a life partner or performing a physically demanding job, having a high score of fatigue or being already physically active at the study entry. There were no significant predictors of group contamination for control group participants.

**TABLE 3:** Predictors of study adherence for the intervention and contamination for the control group.

Predictors	Intervention (*n* = 67)		Control (*n* = 67)	
	Odds ratio (95%‐CI)	*p*‐value	Odds ratio (95%‐CI)	*p*‐value
Gender (female)	2.35 (1.07–5.18)	**0.03**	0.18 (0.02–1.03)	0.08
BMI ≥25 kg/m^2^ (yes)	1.39 (0.82–2.34)	0.22	0.71 (0.11–3.63)	0.70
Having a partner (yes)	1.76 (0.81–3.82)	0.16	1.41 (0.33–6.34)	0.64
Physical demanding job (yes)	1.71 (0.72–4.06)	0.22	0.89 (0.170–4.11)	0.88
Physical component summary (z‐score)	1.34 (1.07–1.69)	**0.01**	0.62 (0.21–1.90)	0.38
Mental component summary (z‐score)	1.37 (1.13–1.66)	**0.001**	0.603 (0.28–1.26)	0.18
Fatigue, checklist individual strength (z‐score)	0.85 (0.65–1.10)	0.22	0.44 (0.11–1.54)	0.21
Baseline PA (h/week)	1.00 (0.99–1.01)	0.71	0.67 (0.330–1.21)	0.22
Intervention week (ascending)	0.98 (0.98–0.99)	**<0.001**	‐	‐

*Note*: Odds ratios and their 95%‐CI as results from logistic regression analyses for study adherence (intervention group) and study contamination (control group). Adherence of intervention group participants was based on reported weekly diary entries through the entire study period (missings set to 0 h exercise/day) and covariates gathered at baseline and updated at T3 (BMI, CIS) and T6 (BMI, PCS, MCS, CIS). Contamination of controls was based on reported weekly intense physical activity hours at baseline and study end (contamination when >60 min weekly exercise improvement).

Abbreviation: BMI, body mass index.

## 4 DISCUSSION

This analysis (*n* = 144, 43% female) investigated the patterns of adherence and contamination in a PA behavior trial and its predictors using the SURfit study, a tailored 1‐year PA behavior RCT for adolescent and adult CCS. Overall, approximately one in two adhered to the PA program, while the program was contaminated by approximately one in six controls. While adherents to the intervention program reached 100% of their PA goal over the whole study period, non‐adherents decreased their PA hours from the very beginning with a clear difference of the exercise behavior from week 4 into the program. Nearly half of the averaged 2.5 h of weekly PA was spent in sport types categorized as jogging, cycling, and swimming. Predictors of PA adherence were less time into the program, female sex, and higher physical and mental quality of life. There were no predictors of contamination of the control group.

To estimate the true treatment effect of an RCT, it is important that the intervention and the control group adhere to their allocated study program. The definition of adherence differs across exercise studies^7^ and makes a comparison difficult.^6^, ^17^, ^18^, ^19^ In our study, around half of intervention participants fulfilled ≥2/3 of the expected training (baseline activity +2.5 h intense PA/week). This threshold was also used by others^6^ and seems to a be a reasonable approach, as it takes true life into account with periods when exercising is not possible, for example, family life with children, vacations, or acute infections. An equally important, but far less acknowledged problem of RCTs aiming to increase PA is contamination of the control condition. Approximately one in six of controls contaminated group allocation in our trial by increasing their intense PA by >60 min/week corresponding to a mean increase from their average baseline PA hours by 62%. These adherence and contamination issues have been consistently described in comparable RCTs (42%–91% for intervention,^6^, ^8^, ^17^, ^18^, ^19^ 22%–52% for control group participants^20^, ^21^) suggesting that compliance issues occur in both groups. These issues may be an important reason why a substantial amount of trials aiming to increase PA end with null findings. By carefully monitoring compliance of control and intervention groups^6^, ^13^, ^21^ as a precondition of each PA trial, we will learn more about who is participating, why, and for how long. This information is valuable and needs to be taken into account when interpreting the effect of such interventions.

PA hours of the intervention group were self‐reported in a web‐based diary, which has been successfully used for assessing adherence in RCTs also in cancer survivors.^1^, ^22^ As self‐reported PA behavior may be hampered by desirability bias, the validity of our participants' entries were checked based on self‐reported and retrospectively extracted pedometer steps with acceptable agreement (data not shown) as found in other research.^23^ Based on the nature of sports performed (e.g., swimming, bicycling), objective measures by pedometers or accelerometers may not be better alternatives as often suggested^7^, ^10^, ^23^ as these types of activities get poorly detected by motion sensors. PA adherents and non‐adherents did proportionally the same type of activities. This indicates that the type of PA was not decisive for participants' adherence. However, while the hourly length of each training session was comparable, adherents performed training sessions on average every second day and thus nearly twice as often as non‐adherents.

We found descending intervention week, female sex, and low physical and mental quality of life to be significant positive predictors for intervention adherence. This is in agreement with most studies that reported a high adherence in short‐term, but a striking loss of adherence in long‐term PA trials.^9^, ^17^ Comparably, we also lost the non‐adherents and dropouts very early. Interestingly, behavioral change was established by the fourth week, from which time point it was possible to distinguish between PA adherents and non‐adherents based on their diary entries. Thus, it is especially important to track participant's PA behavior during the first weeks to be able to interfere and re‐motivate them if needed with a combination of different motivational approaches.^24^ Alternatively, one could consider different study designs such a Single‐Case Experimental Design (SCEDs) in which the relationship between different levels of treatment and changes in behavioral and biological outcomes are measured on an individual that serves as his/her own control.^25^ As an example, everyone would experience his/her preferred PA regimen for the first 2 months and then would be randomized to either the optimal treatment identified during the 2 months or to alternative interventions to be studied, so that dropouts and non‐adherers would not be randomized in an intention‐to‐treat format.

For the control group, no significant predictors for study contamination were found which may be partly explained by the small sample size in the “contaminated” group. Misclassification based on reporting bias cannot be ruled out either. As trials are always prone to a clear selection bias toward those willing and ready to increase their PA behavior, other designs such as SCEDs as mentioned above could be a solution to solve this problem.

Predictors of adherence to the intervention can help to understand adherence in trials focusing on behavioral change, for example, whom we might reach and why. Female compared to male sex is globally associated with poorer PA during adolescence in the healthy population and CCS.^2^, ^22^ Despite this fact of lower PA in the female population in general, no difference in PA hours between sexes were seen in our study at baseline. This could be due to selection bias because females that were willing and motivated to exercise were more likely to participate in the study. Moreover, a stronger association between exercise behavior and regulations among women than men has previously been described, but this was not consistent.^7^, ^22^, ^24^ Participants with reduced physical and mental quality of life adhered less to the PA program. This finding goes along with current research^1^, ^10^ documenting that those with physical limitations from cancer therapy or mental health‐related disorders had a lower ability to fully take part in and adhere to the intervention. It is therefore important that major efforts are taken at the start of the program to convince this population in taking up PA from the very beginning and make them feel physically and mentally better. Mainly due to a constant decrease in the non‐adherent group, PA adherence decreased from the first to the second half of the intervention period. This is in line with other research documenting higher adherence in short‐term than in long‐term studies.^9^, ^17^ Other potential predictors of adherence such obesity, lack of social support, high physical work load, fatigue, or different baseline PA documenting resistance or inability to increase PA, were not predictive of adherence in our study.

### 4.1 Strengths and limitations

Strengths of our study include the RCT design and the long duration of a 1‐year individualized program. The schedule was embedded in each participant's daily living, allowing greatest flexibility in the planning and performing of PA as well as sustained behavioral change to take place. The PA program was followed‐up by regular phone calls and meetings by trained physiotherapists and supported by various motivational features. In parallel, long‐term adherence was tracked which is much more relevant than short‐term adherence.^7^ Finally, we looked also at contamination issues that are rarely assessed in PA behavioral change trials.

A limitation in all trials focusing on change in PA behavior is the highly selective and mostly sport‐motivated participants, especially in CCS where survivors with mental or physical handicaps related or not related to the cancer history tend not to participate.^2^ A further limitation is the restricted sample size calculated for the primary outcome of SURfit (cardiovascular health), although this is one of the largest published exercise trials focusing on CCS. This has limited the power and the maximal number of predictors to analyze especially in the control group. Besides, self‐reported PA hours are prone to social desirability bias leading to over‐reporting and misclassification.^13^ PA assessment in the control group was only assessed at baseline and at 12 months in order to minimize contamination, but at the expense of a possible reporting bias. Whether repeated assessments of cumulative PA hours in the control group is wise or provokes an even larger “contamination” could for instance be determined by two different control groups using different approaches to assess PA.

### 4.2 Practical implications

Our findings can help to improve future PA behavioral interventions. Trials should be aware of non‐adherence, especially in the beginning of a behavioral intervention. Our results showed that the attitude towards behavioral change was established in the first month of the intervention. Furthermore, contamination needs to be considered in studies that recruit participants willing or motivated to enhance their PA. Power calculations are a critical first step when a trial is planned; expected effect sizes could be modeled on the assumption of a lower increase in PA than expected among the intervention group because of non‐adherence, a parallel increase in PA among controls, and dropouts thereby reducing the actual delta between the two arms. Taking our findings of non‐adherence and contamination together, alternative study designs like attention control groups, SCEDs or step‐wedged designs could be valuable alternative options. One also needs to be aware of the problem of self‐selection bias in PA behavior interventions, particularly in studies of survivors where strategies should be applied to screen and potentially exclude already highly active participants.

### 4.3 Conclusion

Adherence to the intervention, prevention of contamination in the control group and the assessment of their predictors are crucial to interpret efficacy and clinical relevance of PA behavior RCTs. Even with high motivational support, only half of the intervention group adhered to their expected exercise regimen, while one in six controls contaminated the study by increasing their PA level beyond 1 h/week. While exercise adherents reached about 100% of their individual goal during the whole study, non‐adherents reduced their weekly PA hours already after the first months and remained there. Assessing PA levels at this time could identify participants that could benefit from more strenuous supervision and advice. This would accurately reflect the goal of properly enabling everyone to participate in these valuable research studies. Our results raise awareness about the problem of non‐adherence and contamination in behavioral trials. These problems should be considered when interpreting results of comparable trials; they should be part of any sample size calculation when a behavioral intervention is planned; and they may even be indicative for using alternative study designs such as SCEDs or step‐wedge designs where optimal treatments based on individual interventions and proper monitoring of PA could be reached, while minimizing non‐adherence and contamination.

## AUTHOR CONTRIBUTIONS

**Ruedi Jung:** Conceptualization (supporting); data curation (supporting); formal analysis (lead); investigation (supporting); methodology (supporting); project administration (supporting); visualization (lead); writing – original draft (lead); writing – review and editing (lead). **Simeon Joel Zuercher:** Conceptualization (supporting); data curation (supporting); formal analysis (supporting); investigation (supporting); methodology (supporting); project administration (supporting); visualization (supporting); writing – original draft (supporting); writing – review and editing (supporting). **Christina Schindera:** Project administration (supporting); writing – original draft (equal); writing – review and editing (supporting). **Julia Braun:** Data curation (supporting); formal analysis (supporting); writing – review and editing (supporting). **Wei Hai Deng:** Writing – review and editing (supporting). **Nicolas X. von der Weid:** Conceptualization (supporting); funding acquisition (supporting); investigation (supporting); supervision (supporting); writing – review and editing (supporting). **Corina Silvia Rueegg:** Conceptualization (lead); data curation (supporting); formal analysis (supporting); funding acquisition (lead); investigation (supporting); methodology (lead); project administration (supporting); supervision (supporting); writing – original draft (supporting); writing – review and editing (supporting). **Susi Kriemler:** Conceptualization (lead); formal analysis (supporting); funding acquisition (lead); investigation (supporting); methodology (lead); project administration (supporting); resources (supporting); supervision (lead); visualization (supporting); writing – original draft (supporting); writing – review and editing (supporting).

## FUNDING INFORMATION

Swiss Cancer League (KLS‐3175‐02‐2013), “Stiftung für krebskranke Kinder, Regio Basiliensis,” “Gedächtnis‐Stiftung Susy Rückert zur Krebsbekämpfung,” “Taecker‐Stiftung für Krebsforschung,” “Stiftung Henriette&Hans‐Rudolf Dubach‐Bucher,” “Stiftung zur Krebsbekämpfung,” “Stiftung Krebs‐Hilfe Zürich,” “Fondation Recherche sur le Cancer de l'Enfant (FORCE),” Fond'Action contre le Cancer, and South‐Eastern Norway Regional Health Authority (project number 2019039). CSR has received funding from European Union Seventh Framework Programme (FP7‐PEOPLE‐2013‐COFUND, grant agreement no 609020‐Scientia Fellows). WHD is paid by a research grant from the South‐Eastern Norway Regional Health Authority (grant number 2019039, to CSR).

## CONFLICT OF INTEREST STATEMENT

None.

## Supporting information


Figure S1.

## Data Availability

Data sharing is not applicable to this article as no new data were created or analyzed in this study.
