# Supplementary material for: Adherence and contamination in a 1‐year physical activity program in childhood cancer survivors: A report from the SURfit study
Source: Cancer Med. 2023 May 18;12(13):14731–41. doi: 10.1002/cam4.6096 (PMC10358195; doi:10.1002/cam4.6096)
Supplement: Supplementary file 1 — Figure S1. [file CAM4-12-14731-s001.pptx]

## Slide 1
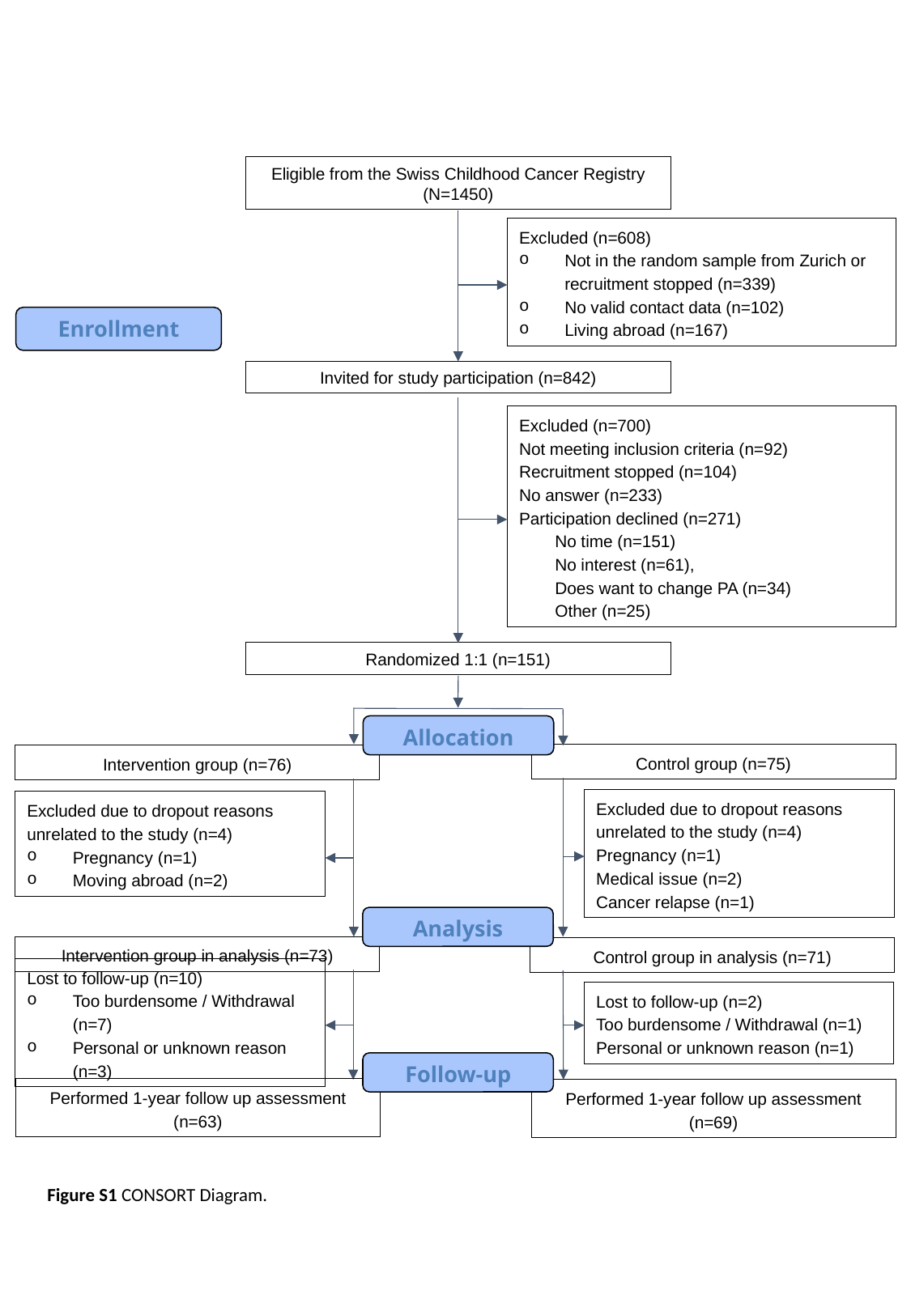

Eligible from the Swiss Childhood Cancer Registry (N=1450)
Excluded (n=608)
Not in the random sample from Zurich or recruitment stopped (n=339)
No valid contact data (n=102)
Living abroad (n=167)
Enrollment
Invited for study participation (n=842)
Excluded (n=700)
Not meeting inclusion criteria (n=92)
Recruitment stopped (n=104)
No answer (n=233)
Participation declined (n=271)
No time (n=151)
No interest (n=61),
Does want to change PA (n=34)
Other (n=25)
Randomized 1:1 (n=151)
Allocation
Control group (n=75)
Intervention group (n=76)
Analysis
Intervention group in analysis (n=73)
Performed 1-year follow up assessment (n=63)
Performed 1-year follow up assessment (n=69)
Excluded due to dropout reasons unrelated to the study (n=4)
Pregnancy (n=1)
Medical issue (n=2)
Cancer relapse (n=1)
Excluded due to dropout reasons unrelated to the study (n=4)
Pregnancy (n=1)
Moving abroad (n=2)
Control group in analysis (n=71)
Lost to follow-up (n=10)
Too burdensome / Withdrawal (n=7)
Personal or unknown reason (n=3)
Lost to follow-up (n=2)
Too burdensome / Withdrawal (n=1)
Personal or unknown reason (n=1)
Follow-up
Figure S1 CONSORT Diagram.
